# A protocol for a randomized trial evaluating the role of carbon‐ion radiation therapy plus camrelizumab for patients with locoregionally recurrent nasopharyngeal carcinoma

**DOI:** 10.1002/cam4.6742

**Published:** 2024-01-11

**Authors:** Jiyi Hu, Qingting Huang, Weixu Hu, Jing Gao, Jing Yang, Haojiong Zhang, Jiade Jay Lu, Lin Kong

**Affiliations:** ^1^ Department of Radiation Oncology, Shanghai Proton and Heavy Ion Center Fudan University Cancer Hospital Shanghai China; ^2^ Department of Radiation Oncology Shanghai Proton and Heavy Ion Center Shanghai China; ^3^ Shanghai Key Laboratory of radiation oncology Shanghai China; ^4^ Shanghai Engineering Research Center of Proton and Heavy Ion Radiation Therapy Shanghai China; ^5^ Department of Radiation Oncology, Proton and Heavy Ion Center Heyou International Hospital Foshan China

**Keywords:** camrelizumab, carbon ion, radiotherapy (RT), randomized trial, recurrent nasopharyngeal carcinoma

## Abstract

**Purpose:**

Management of locoregionally recurrent nasopharyngeal carcinoma (LR NPC) is difficult. Although carbon‐ion radiation therapy (CIRT) could substantially improve the overall survival (OS) of those patients, around 40% of the patients may still develop local failure. Further improvement of the disease control is necessary. Immunotherapy, such as immune checkpoint inhibitors (ICIs) becomes a promising antitumor treatment. The role of ICIs was proved in head and neck cancers including recurrent/metastatic NPC. Preclinical studies indicated potential synergistic effects between radiation therapy and ICIs. Therefore, we conduct a randomized phase 2 trial to evaluate the efficacy and safety of camrelizumab, an anti‐PD‐1 monoclonal antibody, along with CIRT in patients with LR NPC.

**Methods:**

Patients will be randomly assigned at 1:1 to receive either standard CIRT with 63 Gy (relatively biological effectiveness, [RBE]) in 21 fractions, or standard CIRT plus concurrent camrelizumab. Camrelizumab will be administered intravenously with a dose of 200 mg, every 2 week, for a maximum of 1 year. We estimate addition of camrelizumab will improve the 2‐year progression‐free survival (PFS) from 45% to 60%. A total of 146 patients (with a 5% lost to follow‐up rate) is required to yield a type I error of 0.2, and a power of 0.8.

**Results and Conclusion:**

The results of the trial may shed insights on the combined therapy with ICIs and CIRT.

## 1 INTRODUCTION

Clinical treatment for locoregionally recurrent nasopharyngeal carcinoma (LR NPC) is challenging. Currently, endoscopic surgery and reirradiation using intensity‐modulated radiation therapy (IMRT) are the mainstay of treatment for LR NPC. Surgery may provide good local control in carefully selected patients with relatively early stage disease.^1^ A randomized trial showed that endoscopic nasopharyngectomy could significantly improve the overall survival (OS) in highly selected patients (with relatively early stage), the corresponding 3‐year OS rate was 85.5%.^1^ However, the strict indication and requirement of surgeon's expertise limits the wide application of endoscopic surgery in LR NPC. Reirradiation by IMRT remains one of the options as salvage treatment, and it is the only curative treatment in patients with locally advanced diseases. Nevertheless, the clinical outcome after IMRT using conventional fractionation is unsatisfactory, probably due to the severe toxicities induced by reirradiation and the radioresistance of the recurrent tumor cells. Previous reported 2‐year OS rates were around 50%–60%.^2^, ^3^ Hyperfractionated IMRT was able to significantly improve the OS to over 70%, although the locoregional relapse‐free survival rate remains at a relatively low level of 53.7%.^4^

Carbon‐ion radiation therapy (CIRT) is a newly emerging technique with both physical and biological advantages.^5^ Physically, the highly conformal dose distribution of carbon‐ion beam enables better protection of surrounding organs at risk (such as temporal lobe and brain stem).^6^ Biologically, carbon‐ion beam causes complex double‐strand breakage of DNA that is more difficult to repair, thus leading to efficient killing of resistant tumor cells. The physical and biological features of the CIRT confers potential benefits in the treatment of LR NPC. The clinical studies also indicated a possible improvement in OS.^7^, ^8^ Previously, we reported the clinical outcome of 206 patients with LR NPC treated using CIRT.^8^ The outcome was promising with a 2‐year OS rate of 83.7%. Meanwhile, the incidence of mucosal necrosis and subsequent lethal hemorrhage dropped to 16% and <10%, respectively. However, there were still around 40% of the patients developed local failure 2 years after CIRT. The outcome of those patients are dismal. Improvement of the disease control is in need.

Immune checkpoint inhibitors (ICI), such as anti‐programmed cell death‐1 (PD‐1) andtibodies, have been extensively studied in recurrent/metastatic head and neck squamous cell carcinoma (R/M HNSCC) including NPC.^9^, ^10^, ^11^ In the CAPTAIN‐1st (phase 3) trial, Yunpeng Yang et al. showed that addition of cmarelizumab to gemcitabine and cisplatin (GP) could significantly improve the progression‐free survival (PFS) for patients with R/M NPC (hazard ratio [HR], 0.54; *p* = 0.0002).^12^ Similar results were found in the JUPITER‐2 trial conducted by Haiqing Mai and his colleagues.^13^ In this randomized phase 3 trial, a significant improvement in PFS was observed in R/M NPC patients treated with toripalimab plus GP, compared to GP alone (HR, 0.52; *p* = 0.0003). Preclinical studies showed that radiotherapy could alter the tumor microenvironment thus providing a potential synergistic effect when used concurrently with ICIs.^14^, ^15^ And CIRT could induce similar or even stronger proinflammatory effects, compared to X‐ray radiotherapy.^16^, ^17^, ^18^, ^19^ Clinically, the combined effects of chemoradiotherapy and ICIs have been demonstrated in non‐small cell lung cancer (NSCLC).^20^, ^21^ In the PACIFIC trial, the authors demonstrated the benefit from combined treatment of chemoradiotherapy plus durvalumab, compared to chemoradiotherapy alone, in both PFS (HR, 0.52; *p* < 0.001) and OS (HR, 0.68; *p* = 0.0025).^21^

However, the role of ICIs used in concurrent with radiation therapy in LR NPC has not been evaluated. This proposed randomized phase 2 clinical trial is to examine the efficacy and toxicity profile of radiation therapy with concurrent camrelizumab, a IgG4‐kappa PD‐1 monoclonal antibody, in patients with LR NPC.

## 2 METHODS

### 2.1 Patients eligibility

This study will recruite patients with LR NPC treated at Shanghai Proton and Heavy Ion Center (SPHIC). Patients are eligible if they: (1) were previous diagnosed as NPC by pathology; (2) are pathologically and/or clinically (via MRI) confirmed of local or locoregional recurrence; (3) have completed a definitive course of radiation therapy to the nasopharynx (a total dose ≥66Gy to the gross tumor volume); (4) have a disease‐free interval of ≥6 months after the completion of the first course of radiation therapy; (5) are feasible for definitive treatment; (6) have an age of 18–70 year‐old; (7) are able to receive magnetic resonance (MR) scans; (8) have an ECOG score < =1; (9) have sufficient major organ function.

The exclusion criteria include: distant metastasis; severe late toxicities of prior radiation therapy; previous radioactive particle implantation within the treatment field; previous malignancy except for squamous/basal cell carcinoma of the skin, or cervical carcinoma in situ; previous use of ICIs (such as anti‐PD‐1/L1, or CTLA‐4 inhibitors), or other immunotherapy targeting T‐cell costimulating; pregnancy or lactation; active noninfectious penumonia; immunodeficiency virus; active hepatitis B or C infection; previous pneumonitis; autoimmune disease, or immunosuppression; medical conditions requiring use of steroids or other immunosuppressive medications; previous application of high dose systemic steroid within 14 days before initiation of treatment; use of antibiotics for a duration ≥5 days within 1 month before initation of camrelizumab.

### 2.2 Treatment groups

Patients will be randomized at 1:1 to control group and experimental group using a web‐based platform. The randomization is stratified by recurrent stage (stage 1/2 vs. stage 3/4) according to the American Joint Committee on Cancer (AJCC) staging manual (the 8th edition).

All patients in both groups will receive CIRT with the same regimen. Patients are fixed at the supine position using the head, neck, and shoulder mask and alpha cradle. Simulation CT scans with a 1.5 mm thickness are then performed. Contrast‐enhanced MR scans are Simulation CT scans and contrast‐enhanced MR scans at the same position are then performed and fused to guide targets delineation. The gross tumor volume (GTV) defined as the disease detected on the imaging studies. A 5 mm margin is added to GTV to generate clinical target volume (CTV). And CTV is exapanded by a 3–6 mm margin (depending on the surrounding organs at risk [OARs]) to generate planning target volume. The dose constraints for CIRT were detailed in Table S1. And a recovery rate of 70% from previous irradiation was assumed based on the research by Nieder and our previous experience.^7^, ^8^, ^22^ The total dose to GTV is 63 Gy (relative biological effectiveness, RBE) in 21 fractions. This is the recommended dose from our phase 1 trial.

**FIGURE 1:**
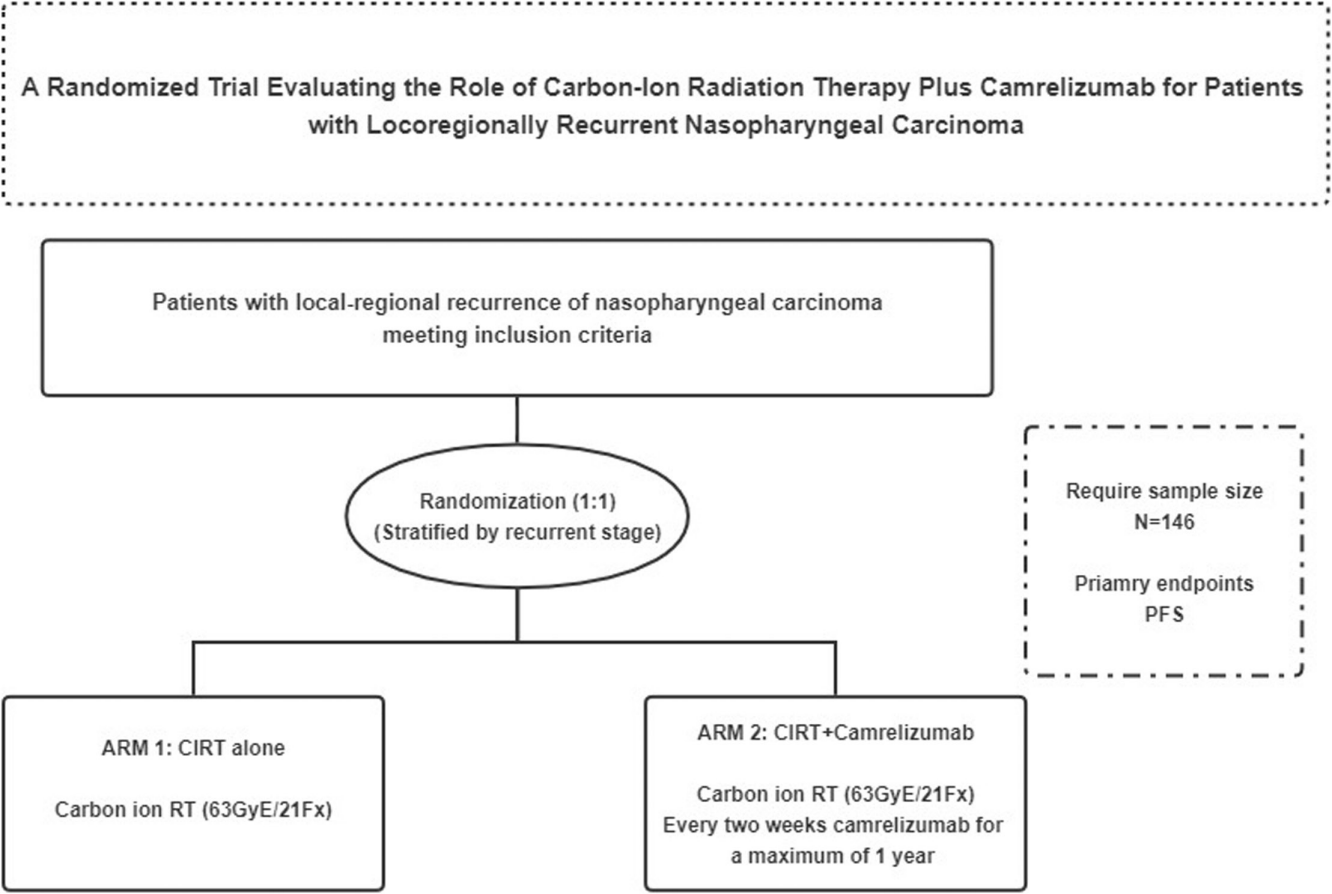
Study scheme.

Patients in the experimental group will receive camrelizumab intravenously starting concurrently with CIRT, for a maximum of 1 year. Camrelizumab should be paused for patients who develop Grade ≥2 toxicities related to immunotherapy, or Grade 1 penumonia. Camrelizumab will be resumed if the related toxicities resolve to Grade 1 or baseline, except that patients may resume camrelizumab in the presence of Grade 2 fatigue or skin toxicities. Camrelizumab should be stopped if: the patients develop untolerated toxicities; camrelizumab has been delayed for 3 months because of mild‐to‐moderate toxicities; disease progression (See Figure 1—Study Schema).

Patient may receive 2–3 cycles of induction chemotherapy before CIRT with or without camrelizumab. Gemcitabine plus cisplatin or nedaplatin is recommended but not mandatory. The choice of chemotherapy regimen is at the discretion of the physicians.

### 2.3 Assessment

Before enrollment, patients should be evaluated for history, physical examination, 12‐lead electrocardiogram, echocardiogram, laboratory tests (i.e., complete blood count [CBC], blood chemistry, hepatitis B/C antibodies and DNA copy number, HIV antibody, TSH, free T3/T4, amylase, cardiac enzymes, and coagulation test), and urine and stool test. Extension of the disease will be assess by MR scans of the head and neck, and whole‐body positron emission tomography (PET)‐computed tomography (CT) (could be replaced by plain chest CT, abdominal ultrasound, and bone scan). Patients will be staged according to the AJCC staging manual (the 8th edition). Patients with cervical lymphadenopathy should receive neck dissection before enrollment.

During the treatment, patients will receive weekly evaluation of the toxicities. Toxicities related to camrelizumab will be evaluated every 2 weeks before administration of the drug during the maintenance phase. Regular follow‐up will be arranged at 1 month after CIRT/camrelizumab miantenance and every 3 months thereafter after. At each visit, MR scan of the nasopharynx is scheduled. Patients will also plain chest CT, abdominal ultrasound, and bone scan annually. PET/CT could be prescribed at the discretion of the attending physicians if necessary. Treatment response will be evaluated according to the Response Evaluation Criteria in Solid Tumors (version 1.1).^23^ Toxicities are evaluated according to the Common Terminology Criteria for Adverse Events (version 5.0).

### 2.4 Endpoints

The main purpose of the current study is to assess the efficacy and toxicities of adding camrelizumab to standard CIRT. The primary endpoint is the PFS, which is defined as the time frame from randomization to death or disease progression. The secondary endpoints include overall survival (OS), local progressoin‐free survival (LPFS), regional progression‐free survival (RPFS), distant metastasis‐free survival (DMFS), and toxicity profile. OS, LPFS, RPFS, and DMFS are defined as time frames from randomization to death or local progresion, regional progression, and distant metastasis, respectively. The authors will also explore the potential biomarkers for treatment outcome based on omics.

### 2.5 Statistical considerations

According to the previous studies, the 2‐year PFS rate after CIRT is around 45%. It is estimated that addition of camrelizumab could improve the 2‐year PFS rate to 60%. A total of 146 (with a lost to follow‐up rate of 5%) patients are required to yield a type I error rate of 0.20 and a power of 0.8. The baseline characteristics and efficacy analyses will be performed in the intention‐to‐treatment population. Safety analyses will be performed in patients who received randomly assigned treatments (as‐treated population). PFS, OS, LPFS, RPFS, and DMFS will be calculated using Kaplan–Meier method. Cox proportional hazard model will be used to estimate the hazard ratio.

### 2.6 Ethical considerations and data management

The current trial is approved by the institutional review board (IRB) of SPHIC (approval number: 1906‐33‐02‐2011A) and registered in the ClinicalTrials.gov (ID: NCT04143984). This trial will be conducted according to the Good Clinical Practice guidelines of China and the principles of the Declaration of Helsinki. The conduct of the trial will be monitored by the IRB to ensure the quality of the trial and to protect the rights of the participants. Written informed consent were obtained for patients and researcher before the enrollment of clinical trial.

## 3 DISCUSSION

Various treatment modalities have been attempted to salvage recurrent nasopharyngeal carcinoma failed previous definitive radiotherapy.^24^ In selected patients with early disease, endoscopic surgery could provide excellent clinical outcome with a 3‐year OS rate of 85.8%.^1^ Brachytherapy and stereotactic radiation therapy could also grant acceptable disease control for LR‐NPC with limited extension.^25^, ^26^ Reirradiation with IMRT remains one of the major treatment modalities, especially for patients with advanced disease. The outcome after IMRT using conventional fractionation was not satisfactory with a high incidence of severe RT‐induced toxicities. A large series showed the 5‐year OS rate was 44.9% after IMRT.^27^ RT‐induced injuries accounted for 69.2% of the patients' death. It is postulated that patients who failed previous IMRT (compared to 2DRT) may have a worse outcome. In series of 77 patients who failed previous definitive IMRT, the 3‐year OS rate was 51.5%, and 52.9% of the patients' death was caused by severe toxicities secondary to reirradiation.^3^ Hyperfractionation may serve as a better IMRT scheme in the reirradiation setting. Recently, a randomized trial showed that hyperfractionated IMRT could improve the 3‐year OS to 74.6% by significantly reducing the RT‐induced toxicities.^4^

CIRT, as an advanced radiation technique, could provide better clinical outcome for this group of patients due to its physical and biological advantages. Previously, we have summarized the clinical outcome of 206 LR NPC patients treated using CIRT.^8^ The 2‐year OS, local control, regional control, and distant control rates were 83.7%, 58.0%, 87.3%, and 94.7%. Despite the improved OS compared to historical data of photon‐based IMRT, a proportion of patients suffers from locoregional and distant failure. Further improvement in the disease control is necessary.

ICIs emerges as a promising antitumor therapy. The outcome after ICIs therapy in R/M HNSCC were encouraging.^12^, ^13^, ^28^, ^29^, ^30^, ^31^ Both nivolumab and pembrolizumab showed superiority in patients with platinum‐refractory R/M HNSCC.^28^, ^29^ In the pivotal CheckMate‐141 trial, Ferris et al. showed in 361 patients that nivolumab had significantly improved the OS of R/M HNSCC (HR, 0.70; *p* = 0.01), compared to standard of care chemotherapy (methotrexate, docetaxel, or cetuximab).^29^ In a following trial (KEYNOTE‐040) of 495 patients with similar setting, a significant benefit was also detected for pembrolizumab.^28^ In this trial, Cohen and his colleagues showed that pembrolizumab was associated with a 20% reduction in risk of death (HR, 0.80; *p* = 0.0161). The role of ICIs as first‐line treatment is also demonstrated.^31^ In the KEYNOTE‐048 trial, Burtness et al., showed that, compared to cetuximab with chemotherapy, pembrolizumab alone was associated with significantly improved OS in R/M HNSCC patients with a PD‐L1 combined positive score (CPS) of ≥20 (HR, 0.61; *p* = 0.0007) and patients with a CPS of ≥1 (HR, 78; *p* = 0.0086), while pembrolizumab with chemotherapy was superior in the overall population (HR, 0.77; *p* = 0.0034). The efficacy of ICIs was also specifically tested in R/M NPC. Both the CAPTAIN‐1st trial and JUPITER‐2 trial have demonstrated that R/M NPC patients could benefit from addition of ICIs (camrelizumab or toripalimab) to GP chemotherapy.^12^, ^13^ In the CAPTAIN‐1st trial, a total of 263 patients were enrolled and randomized at 1:1 to camrelizumab with GP or GP. After a median follow‐up time of 15.6 months, camrelizumab with GP was associated with superior PFS (HR, 0.54; *p* = 0.0002), the corresponding median OS were 10.8 months versus 6.9 months favoring camrelizumab with GP; the OS at that ime was immature, thus not analyzed.

Preclinical studies have suggested potential synergistic effects between immunotherapy and radiation therapy. Irradiation could release neoantigens, induce expression of type I interferon and other proinflammatory mediators, upregulate PD‐L1, and modulate tumor microenvironment, thus reinvigorating the host's immune system.^32^, ^33^, ^34^ The efficacy of combined therapy with ICIs and radiation therapy has been explored clinically. The PACIFIC trial was designed to evaluate the efficacy and safety of durvalumab combined with chemoradiotherapy in stage III NSCLC.^21^ With a total of 713 patients enrolled in this trial, the authors showed that compared to chemoradiotherapy, addition of durvalumab was associated with significantly improved PFS (HR, 0.52; *p* < 0.001) and OS (HR, 0.68; *p* = 0.0025), and the corresponding 12‐month PFS and 24‐month OS rates were 55.9% versus 35.3%, and 66.3% versus 55.6% favoring combined therapy. CIRT could exert similar or even greater immunomodulatory effects. In a preclinical study, Spina et al. compared the immunomodulatory ability of photon therapy and CIRT.^18^ The results showed that compared to photon therapy, low dose CIRT was able to spare more lymphocytes, while high dose CIRT could induce higher expression of proinflammatory cytokines.^18^ In another study, Permata et al. showed that CIRT could induce a greater upregulation of PD‐L1 than did the photon therapy, thus having potentially stronger synergistic effects with ICIs.^19^

To the best of our knowledge, the current study is the first clinical trial evaluating the efficacy of ICIs combined with CIRT as salvage treatment for patients with LR NPC. The results of the may shed insights on the combined therapy with ICIs and particle radiation therapy.

## AUTHOR CONTRIBUTIONS

**Jiyi Hu:** Conceptualization (equal); data curation (equal); formal analysis (equal); funding acquisition (equal); investigation (equal); project administration (equal); software (equal); supervision (equal); writing – original draft (equal); writing – review and editing (equal). **Qingting Huang:** Conceptualization (equal); data curation (equal); investigation (equal); writing – original draft (equal). **Weixu Hu:** Investigation (equal); resources (equal); validation (equal); visualization (equal). **Jing Gao:** Data curation (equal); formal analysis (equal); resources (equal); validation (equal); visualization (equal). **Jing Yang:** Data curation (equal); formal analysis (equal); investigation (equal); methodology (equal); resources (equal); software (equal); validation (equal); visualization (equal). **Haojiong Zhang:** Data curation (equal); formal analysis (equal); methodology (equal). **Jiade Jay Lu:** Conceptualization (equal); methodology (equal); project administration (equal); resources (equal); supervision (equal); validation (equal); visualization (equal); writing – review and editing (equal). **Lin Kong:** Conceptualization (equal); data curation (equal); funding acquisition (equal); methodology (equal); project administration (equal); supervision (equal); validation (equal); visualization (equal); writing – original draft (equal); writing – review and editing (equal).

## FUNDING INFORMATION

This work was mainly supported by the Key research and development Program of the Ministry of Science and Technology (project number 2022YFC2401505), the Science and Technology Development Fund of Shanghai Pudong New Area (project number PKJ2022‐Y53), and ShanghaiMunicipal Health Commission Clinical research project (project number 202340150) .

## CONFLICT OF INTEREST STATEMENT

The authors declare no conflicts of interest related to this study.

## ETHICS STATEMENT

This study was approved by the Institution Review Board (IRB) of the Shanghai Proton and Heavy Ion Center, Shanghai, China. (Approval number: 1906‐33‐02‐2011A).

## Supporting information


Table S1

## Data Availability

The datasets generated and/or analyzed during the current study are available from the corresponding author on reasonable request.
