# Supplementary material for: A protocol for a randomized trial evaluating the role of carbon‐ion radiation therapy plus camrelizumab for patients with locoregionally recurrent nasopharyngeal carcinoma
Source: Cancer Med. 2024 Jan 11;13(3):e6742. doi: 10.1002/cam4.6742 (PMC10905325; doi:10.1002/cam4.6742)
Supplement: Supplementary file 1 — Table S1 [file CAM4-13-e6742-s001.docx]

**Supplementary table 1. Dose constraints for organs at risk.**

| **Organs at risk** | **Dose constraint** |
| --- | --- |
| Temporal lobe | D1cm^3^ < 59 Gy RBE |
| Brainstem | D0.1cm^3^ < 46 Gy RBE  D0.7cm^3^ < 38 Gy RBE |
| Optic apparatus | D1% ≤ 50 Gy RBE  D20% ≤ 40 Gy RBE |
